# Functional signaling pathway analysis of lung adenocarcinomas identifies novel therapeutic targets for *KRAS* mutant tumors

**DOI:** 10.18632/oncotarget.5941

**Published:** 2015-09-30

**Authors:** Elisa Baldelli, Guido Bellezza, Eric B. Haura, Lucio Crinó, W. Douglas Cress, Jianghong Deng, Vienna Ludovini, Angelo Sidoni, Matthew B. Schabath, Francesco Puma, Jacopo Vannucci, Annamaria Siggillino, Lance A. Liotta, Emanuel F. Petricoin, Mariaelena Pierobon

**Affiliations:** ^1^ Center for Applied Proteomics and Molecular Medicine, George Mason University, Manassas, VA, USA; ^2^ Medical Oncology Division, S. Maria della Misericordia Hospital, Perugia, Italy; ^3^ Department of Experimental Medicine, Section of Anatomic Pathology and Histology, Medical School, University of Perugia, Perugia, Italy; ^4^ Department of Thoracic Oncology, H. Lee Moffitt Cancer Center and Research Institute, Tampa, FL, USA; ^5^ Department of Thoracic Surgery, University of Perugia, Perugia, Italy

**Keywords:** signaling networks, KRAS mutation, laser capture microdissection, reverse phase protein microarray, non-small cell lung cancers

## Abstract

Little is known about the complex signaling architecture of KRAS and the interconnected RAS-driven protein-protein interactions, especially as it occurs in human clinical specimens. This study explored the activated and interconnected signaling network of *KRAS* mutant lung adenocarcinomas (AD) to identify novel therapeutic targets.

Thirty-four *KRAS* mutant (MT) and twenty-four *KRAS* wild-type (WT) frozen biospecimens were obtained from surgically treated lung ADs. Samples were subjected to laser capture microdissection and reverse phase protein microarray analysis to explore the expression/activation levels of 150 signaling proteins along with co-activation concordance mapping. An independent set of 90 non-small cell lung cancers (NSCLC) was used to validate selected findings by immunohistochemistry (IHC).

Compared to *KRAS* WT tumors, the signaling architecture of *KRAS* MT ADs revealed significant interactions between KRAS downstream substrates, the AKT/mTOR pathway, and a number of Receptor Tyrosine Kinases (RTK). Approximately one-third of the *KRAS* MT tumors had ERK activation greater than the WT counterpart (p<0.01). Notably 18% of the *KRAS* MT tumors had elevated activation of the Estrogen Receptor alpha (ER-α) (*p*=0.02). This finding was verified in an independent population by IHC (*p*=0.03).

*KRAS* MT lung ADs appear to have a more intricate RAS linked signaling network than WT tumors with linkage to many RTKs and to the AKT-mTOR pathway. Combination therapy targeting different nodes of this network may be necessary to treat this group of patients. In addition, for patients with *KRAS* MT tumors and activation of the ER-α, anti-estrogen therapy may have important clinical implications.

## INTRODUCTION

Kirsten rat sarcoma viral oncogene homolog (*KRAS*) is one the most frequent molecular drivers in human cancers and activating mutations of the *KRAS* gene have been found in a wide variety of tumors with greater frequencies in pancreas, colorectal and non-small cell lung cancer (NSCLC) [1]. *KRAS* mutations are found in about 25% of NSCLCs with the highest incidence in the adenocarcinoma (AD) subtype, a subgroup of tumors where up to 30% of patients are affected by the mutation [2]. This study explored the signaling network of *KRAS* mutant (MT) lung ADs to identify therapeutic biomarkers for the development of targeted treatment for this subgroup of patients.

*KRAS* mutations are a negative prognostic factor for NSCLC and a negative predictor of response not only to EGFR tyrosine kinase inhibitors but also to conventional chemotherapy [3-6]. Despite numerous efforts to develop therapeutic agents capable of directly targeting KRAS, this oncogene still represents an undruggable target [7]. Indeed, the absence of allosteric regulatory sites has made the development of compounds against KRAS extremely challenging [8]. Farnesyl transferase inhibitors, a class of compounds targeting a post-translational modification of RAS, have shown little or no benefit in clinical practice [9]. New approaches aiming at modulating the guanine nucleotide binding pocket of G12C *KRAS* MT lesions have been recently proposed, but their clinical efficacy has yet to be proven [8, 10, 11].

Because the constitutive activation of KRAS downstream effectors leads to uncontrolled cell proliferation, selection of targeted therapies for *KRAS* MT patients has often focused on the inhibition of its direct downstream substrates with particular interest in the members of the MAPK signaling pathway [12-14]. *In vivo* and *in vitro* studies have also evaluated the efficacy of targeting *KRAS* MT tumors using combination therapies, a strategy that has currently been tested in clinical trials [13, 15, 16]. Indeed, KRAS is not only a central node in modulating the transduction of a large number of Receptor Tyrosine Kinases (RTK) (including the EGFR family) via the MAPK pathway, it is also involved in elaborate cross-talk with the PI3K/AKT/mTOR pro-survival pathway. For these reasons combination therapy may be needed to successfully inhibit the KRAS signaling network [17-19]. Although a number of genomic and proteomic studies have been conducted over the years to elucidate the effect of *KRAS* mutations on tumor cells [7, 17, 20] in reality, the true nature of the KRAS signaling architecture *in vivo* within the complex tumor host microenvironment has so far been only marginally explored.

Due to the cross-talk between KRAS and a number of different signaling pathways, we hypothesized that the signaling architecture of *KRAS* MT tumors is more complex than in wild-type (WT) lesions. The elucidation *in vivo* of the KRAS network is critical to identify targets that functionally coordinate the signal propagated by and through KRAS. We utilized reverse phase protein microarray (RPPA) technology coupled with laser capture microdissection (LCM) to map the signaling architecture of *KRAS* WT and MT human lung ADs and to evaluate KRAS linkage in human samples.

## RESULTS

Of the 58 samples analyzed by RPPA, 34 were *KRAS* MT and 24 *KRAS* WT. Among the *KRAS* MT samples the proportion of patients with G12C, G12V, G12D, and G13D mutations was 53%, 26%, 12%, and 9% respectively. Differences in the signaling architecture of *KRAS* MT subtypes were not evaluated due to the low number of counts per group (G12C n=18, G12V n=9, G12D n=4, and G13D n=3). Stage distribution was equal between WT and MT samples, while a higher proportion of males was found in the MT group (Table 1A).

**Table 1:** Clinicopathological characteristics of patients analyzed by RPPA (Panel A) and by IHC (Panel B)

Panel A				
Characteristics		*KRAS* MT(n=34)	*KRAS* WT(n=24)	*P* value
**Age** (median and range)	69.4 (48-90)	67.5 (51-88)	72.0 (48-90)	0.1
**Sex** N (%)				
Male	29 (50.0)	21 (61.8)	8 (33.3)	0.03
Female	29 (50.0)	13 (38.2)	16 (66.7)	
**Stage** N (%)				
IA	15 (25.9)	9 (26.5)	6 (25.0)	0.26
IB	19 (32.8)	11 (32.4)	8 (33.3)	
IIA	3 (5.2)	1 (2.9)	2 (8.3)	
IIB	8 (13.8)	3 (8.8)	5 (20.8)	
IIIA	10 (17.2)	9 (26.5)	1 (4.2)	
IIIB	2 (3.4)	1 (2.9)	1 (4.2)	
IV	1 (1.7)	0 (0.0)	1 (4.2)	

Panel B				
Characteristics		*KRAS* MT(n=46)	*KRAS* WT(n=44)	*P* value
**Age** (median and range)	65.4 (40-81)	63.2 (40-80)	67.7 (45-81)	0.01
**Sex** N (%)				
Male	71 (78.9)	36 (78.3)	35 (79.5)	1
Female	19 (21.1)	10 (21.7)	9 (20.5)	
**Histology** N (%)				
Adenocarcinoma	69 (76.7)	36 (78.3)	33 (75.0)	0.96
Squamous	14 (15.5)	7 (15.2)	7 (15.9)	
Large cell carcinoma	5 (5.6)	2 (4.3)	3 (6.8)	
Mixed	2 (2.2)	1 (2.2)	1 (2.3)	
**Stage** N (%)				
IA	34 (37.8)	16 (34.8)	18 (40.9)	0.32
IB	20 (22.2)	10 (21.7)	10 (22.7)	
IIA	7 (7.8)	2 (4.3)	5 (11.4)	
IIB	3 (3.3)	2 (4.3)	1 (2.3)	
IIIA	22 (24.4)	12 (26.1)	10 (22.7)	
IIIB	4 (4.4)	4 (8.7)	0 (0.0)	
IV	0 (0.0)	0 (0.0)	0 (0.0)	

Correlation analysis between KRAS downstream substrates and the expression/activation levels of the 145 analytes measured by RPPA showed an overall more complex network in the *KRAS* MT population with a greater number of correlations reaching statistical significance compared to the WT group (Supplementary Tables 2 and 3). Spearman's Rho correlation coefficients ranged between 0.6 and 0.9 for the statistically significant relationships.

**Figure 1:**
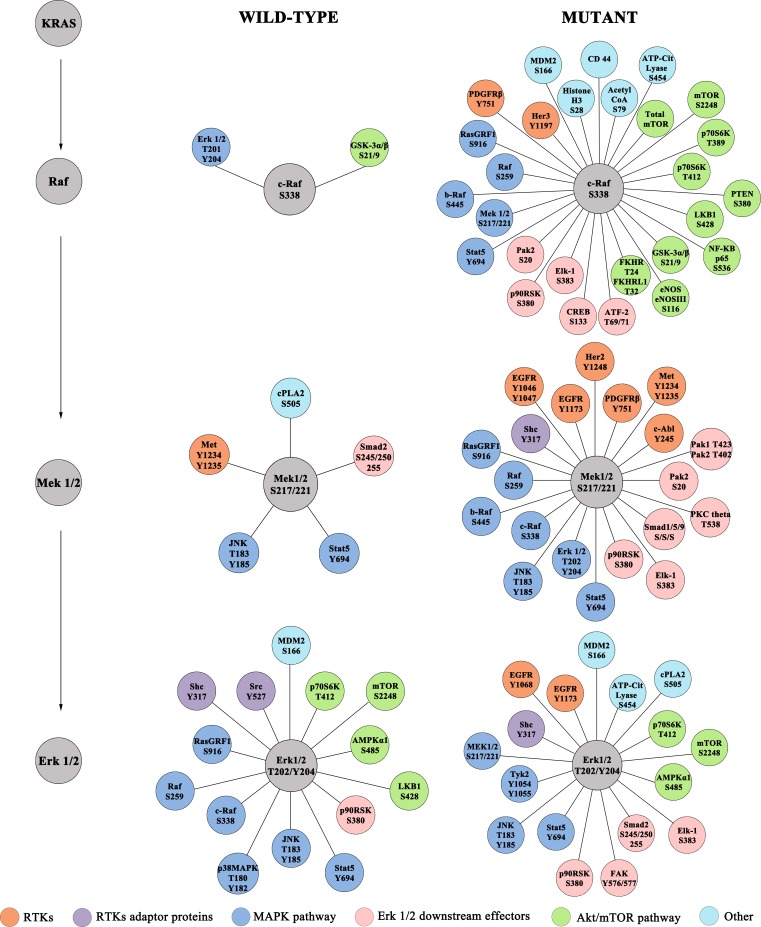
Correlation analysis of KRAS downstream substrates (activated c-Raf, Mek 1/2 and ERK 1/2) and expression/activation levels of 145 endpoints analyzed Only correlations with *p* < 0.0003 are shown.

As expected, significant correlations between the MAPK pathway were almost exclusively found in the *KRAS* MT group (e.g. c-Raf S338 with Mek 1/2 S217/221; Mek 1/2 S217/221 with b-Raf S445, c-Raf S338 and ERK T202/Y204; and finally ERK 1/2 T202/Y204 with Elk-1 S383), which provided confidence in the overall fidelity of the clinical sample analysis (Figure 1). Moreover, in the *KRAS* MT population significant interactions were observed between KRAS downstream effectors and a large number of proteins involved in the AKT/mTOR signaling pathway (Figure 1, Supplementary Tables 2 and 3). Finally the *KRAS* MT cohort showed strong correlations between activated KRAS effectors and several RTKs including phosphorylated EGFR, ErbB2, ErbB3, Ret, and Ron (Figure 1, Supplementary Tables 2 and 3).

**Figure 2:**
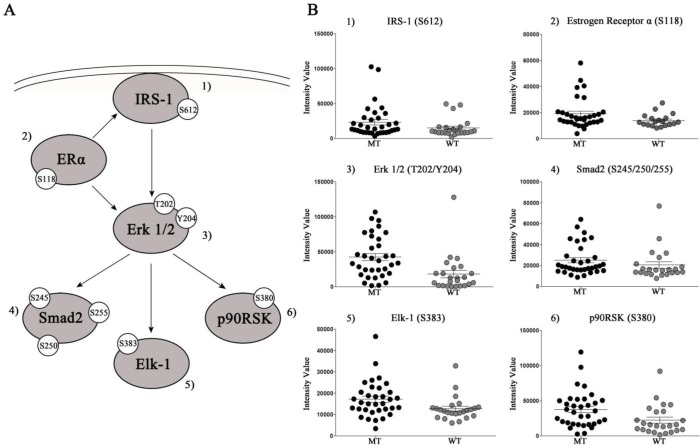
Panel A: Representation of selected proteins that were significantly higher in *KRAS* MT tumors Panel B: Scatter plots of RPPA intensity values with mean and standard error of mean.

We then explored whether the level of activation/expression of the 150 endpoints measured by RPPA was significantly different between *KRAS* WT and MT samples. Eleven analytes reached statistical significance including, as expected, a number of the MAPK family members like ERK 1/2 T202/Y204 and its downstream substrates Elk-1 S383, p90RSK S380, and Smad-2 S245/250/255 (Table 2). Of interest, while approximately one third of the *KRAS* MT samples had very high relative activation of ERK, for the remaining samples the activation level of ERK was comparable between the MT and WT population (Figure 2).

**Table 2:** Analytes found to be statistically different between *KRAS* MT and *KRAS* WT tumors The right column shows the trend in *KRAS* MT group compared to the WT.

Endpoints statistically different	Statistical Test	*P* value	Trend in MT
AMPKα1 (S485)	Wilcoxon rank sum test	0.04	↑
eIF2α (S51)	Wilcoxon rank sum test	0.04	↓
Elk-1 (S383)	Wilcoxon rank sum test	<0.01	↑
ERK 1/2 (T202/Y204)	Wilcoxon rank sum test	<0.01	↑
Estrogen Receptor α (S118)	Wilcoxon rank sum test	0.02	↑
GRB2	Two Sample t-test	<0.01	↓
IRS-1 (S612)	Wilcoxon rank sum test	0.04	↑
p70S6Kinase (T412)	Wilcoxon rank sum test	<0.01	↑
p90RSK (S380)	Wilcoxon rank sum test	<0.01	↑
Smad2 (S245/250/255)	Wilcoxon rank sum test	0.05	↑
Survivin	Two Sample t-test	0.04	↓

Six of the 34 patients (18%) with a *KRAS* mutation showed an increased phosphorylation of the Estrogen Receptor alpha (ER-α) S118 (*p*=0.02), an intracellular receptor that modulates the activation of ERK and other members of the MAPK pathway. Of the six tumors with high activation of the ER-α, two were men and four were women. 66.7% of the patients with activated ER-α had a G12C mutation, while the remaining 33.3% had a G12V mutation. The quantitative range of the ER-α S118 measured by RPPA for these six patients was similar to the level of activation seen in a large population of ER positive breast cancer patients (data not shown).

**Figure 3:**
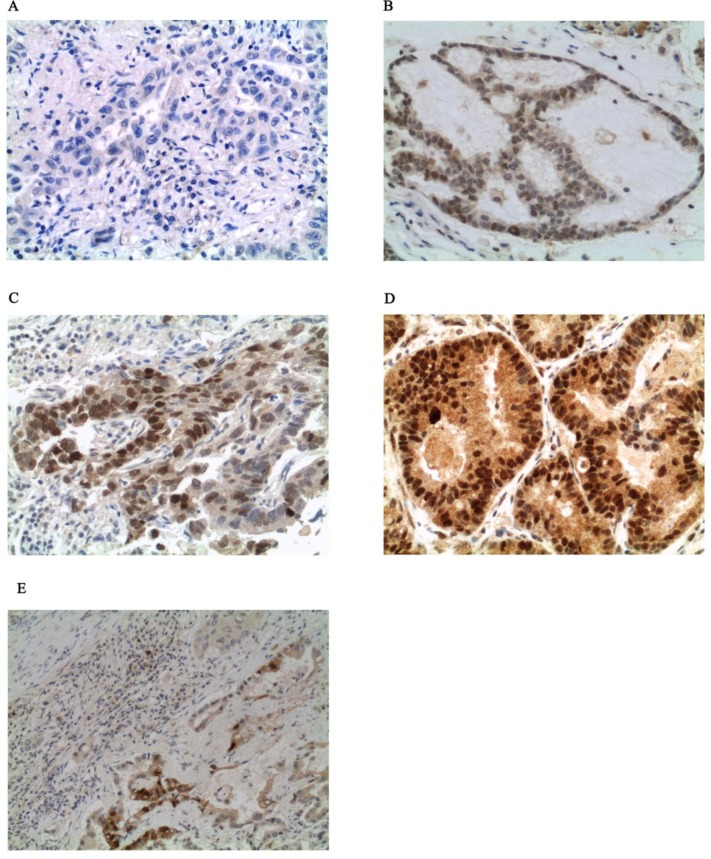
Example of ER-α S118 staining by IHC (x400 magnification) Panel A, B, C, and D show no activation, weak activation (1+), moderate activation (2+), and strong activation (3+) respectively. Panel E shows activation of ER-α S118 in the adenocarcinoma component of the sample with mixed adeno-squamous histology.

Because ER-α is highly targetable by a number of pharmaceutical compounds, to further explore whether ER-α may represent a new drug target for a subgroup of *KRAS* MT lung cancers, we performed IHC analysis on an independent study set of 90 archived NSCLC samples. Of the 90 tumor samples, 46 were *KRAS* MT while the remaining 44 were *KRAS* WT. The validation set included 69 ADs, 5 large cell carcinomas, 14 squamous cell carcinomas and 2 tumors with mixed histology (Table 1 panel B). Within the *KRAS* MT population, the proportion of G12C, G12D, G12V, G12A, G12S, G13C, and G12R mutation was 47%, 22%, 15%, 6%, 4%, 4%, and 2% respectively. Nine of the 46 *KRAS* MT patients (20%) showed ER-α S118 intensity value of 3+ (Figure 3A-3D) while only one patient (2%) in the WT group had similar level of activation of the receptor. All the specimens with intensity scores of 3+ were either ADs or mixed tumors with adeno-squamous components (Table 3). When the analysis was restricted to samples that were pure ADs or mixed adeno-squamous carcinomas, the Pearson's Chi-square test for the intensity scores was highly significant for the *KRAS* MT samples (*p*=0.02). Among the mutant patients 33.3% were female and 66.6% were male. For the 9 *KRAS* MT tumors with high activation of the ER-α, mutations were detected at the sites G12C (66.6%), G12D (22.2%), and G12S (11.1%).

**Table 3:** Distribution of IHC intensity values across the 90 samples analyzed *P* values were calculated using Pearson's Chi-square test.

Intensity value	*KRAS* MT (n=46)	*KRAS* WT(n=44)	Total	*P* value
	N (%)	N (%)		
0	18 (39.1)	16 (36.4)	34	0.034
+	8 (17.4)	15 (34.0)	23	
++	11 (24.0)	12 (27.3)	23	
+++	9 (19.5)	1 (2.3)	10	

Of interest, one of the two cases with mixed histology had a *KRAS* mutation with concomitant ER-α S118 intensity value of 3+. The high activation of ER-α was detected exclusively on the AD component (Figure 3E). Taken together these results provide independent confirmation that the ER-α is highly activated in a subpopulation of *KRAS* MT lung ADs (Table 3). Nonetheless, statistical significance was not reached when the Allred scoring system was applied.

## DISCUSSION

The development of novel therapies to effectively treat NSCLC harbouring *KRAS* mutations is clinically relevant. Because oncogenic *KRAS* has proved to be difficult to target directly, the identification of new druggable targets that are biochemically linked to RAS is of primary importance. The present study is a *de novo* KRAS pathway analysis of carcinoma cells within their native tissue microenvironment.

Because the vast majority of signaling proteins, including those in the KRAS pathway, are ubiquitously expressed and activated to varying extents across different cell types, pure cancer epithelia were first isolated via LCM to minimize the confounding contribution of the tumor-associated stroma. Recently, a number of publications have demonstrated the importance of upfront cellular enrichment when conducting signaling network analysis. For example, using TCGA glioblastoma samples, Mueller and colleagues, have recently shown that the use of LCM is a necessary step to accurately correlate protein expression/activation with genomic alterations (e.g. *PTEN* loss or *EGFR* mutation) [21]. The LCM-RPPA workflow here described provided us with clear and uncontaminated information on the KRAS signaling network of tumor epithelium.

The RPPA platform was selected due to its ability to measure a large number of analytes across hundreds of samples starting from very little biological material [22]. Moreover, this platform has the ability to measure post-translational modification of proteins at relatively low abundance in human cells. Finally, by providing continuous data, this platform has the unique ability to explore the real dynamic range of an analyte of interest. Our quantitative output, as expected, showed that the activation level of ERK 1/2 T202/Y204 (a direct read out of the RAS/Raf/MEK axis), was significantly greater in the *KRAS* MT population compared to the WT population (Figure 2). Nonetheless, the activation level of ERK 1/2 was highly heterogeneous among *KRAS* MT lesions. Phosphorylated ERK 1/2 was significantly higher in approximately one third of the MT population. The remaining MT samples had an ERK 1/2 phosphorylation level comparable to the WT counterpart (Figure 2B). These results indicate that the presence of a *KRAS* mutation is not automatically associated with greater activation of the MAPK pathway. Because pharmacological compounds targeting MEK are under investigation as a potential therapeutic option for *KRAS* MT lung NSCLCs, these data may have important clinical implications. Our exploratory analysis indicates that allocation to anti-MEK targeted treatment based only on *KRAS* mutation status may not be sufficient to identify patients that can benefit from this targeted treatment.

Pair-wise correlation analysis of the 150 key signaling proteins measured by RPPA indicated that the presence of a *KRAS* mutation leads to the formation of distinct linkages between KRAS and a number of signaling pathways. In the MT population the expected members of the MAPK pathway (Mek 1/2 S217/22; ERK 1/2 T202/Y204; Elk-1 S383) and components of the AKT/mTOR pathway showed strong positive interaction with KRAS substrates (Figure 1). A unique set of interactions were detected between proteins directly activated by RAS and members of the pro-survival pathway. In addition significant activation of the T412 site of the mTOR downstream substrate p70S6K was also significantly higher in the *KRAS* MT population, confirming the cross-talk between KRAS signaling and the AKT/mTOR pathway [19]. Finally, KRAS downstream substrates showed significant interaction with phosphorylated PAK1/PAK2 (Supplementary Table 3), a protein kinase involved in a number of different intracellular signaling pathways. Of interest Balbin and colleagues have recently shown that in *KRAS* MT NSCLC cell lines the KRAS-LCK-PAK1/PAK2 axis represents an active network only in KRAS dependent cell lines when compared with the independent counterpart [23]. Due to the lack of biomarkers able to determine KRAS dependency in human tissue, our analysis was able to compare the role of the KRAS-LCK-PAK1/PAK2 axis only between MT and WT lesions. We identified a strong correlation between KRAS and PAK1/2, but not with LCK. Nonetheless the role of PAK1/PAK2 as potential target for *KRAS* MT NSCLC, especially for tumor showing dependency from KRAS, merits further evaluation as potential therapeutic target.

This study also identified a strong linkage between the KRAS signaling network and a number of druggable RTKs (c-Abl Y245, EGFR Y1068 and Y1173; ErbB2 Y1248, ErbB3 Y1197, PDGFR Y751, Ret Y905, Ron Y1353) (Figure 1, Supplementary Tables 2 and 3). Because we specifically analyzed the phospho-specific sites of these RTKs, the correlations found were between KRAS and the functional activation components of these proteins (e.g. phosphorylated RET). Using a proximity ligation assay, Smith and colleagues recently showed that in 25% of surgical specimens collected from NSCLCs harboring a *KRAS* mutations, EGFR associates with the growth factor receptor–bound protein 2 (GRB2) [24]. These findings indicate that the receptor is not only activated, but it is also actively recruiting its down-stream adaptive proteins. RTKs activation in *KRAS* MT ADs merit further evaluation, as it can lead to the identification of new therapeutic targets and to a better understanding of feedback mechanisms established in *KRAS* MT lesions.

These findings indicate that the signaling architecture of *KRAS* MT ADs of the lung is extremely complex and the activity of a number of upstream and downstream KRAS substrates is specifically correlated/modulated by the presence of the mutation. Most likely, the establishment of an intricate network makes *KRAS* MT tumors extremely hard to target and consequently to treat as a population because *in vitro* studies indicated that not all KRAS MT tumors are mutation dependent [23]. Recent *in vitro* proteomic analyses have shown that *KRAS* MT cell lines are also characterized by specific metabolic adaptations. The development of inhibitors against key enzymes involved in these metabolic changes may represent an additional strategy for developing targeted treatment for *KRAS* MT tumors [25]. Due to the complex network that drives *KRAS* tumors, combinatorial multi-targets/multi-pathways inhibitory approach may be necessary to modulate cell growth in patients with a *KRAS* MT NSCLC [26-30].

This analysis identified an unexpected additional potential therapeutic biomarker for ADs of the lung harboring a *KRAS* mutation: the ER-α S118. It is well known that the overall expression of the ER and aromatase are negative prognostic factors in NSCLC [31-34]. The effect of ER on cell growth and differentiation can be carried out using two mechanisms: via classic genomic modulation of transcription factors, or via “non-genomic” action where the ER is involved in intra-cellular cross-talk including a bidirectional interplay with the MAPK signaling pathway, which includes the phosphorylation of the S118 residue [35-38]. Phosphorylation of the S118 induces activation of the receptor and it modulates transcriptional activity, receptor degradation, and response to tamoxifene-based treatment [39].

While a number of studies have evaluated the expression and activation of the ER (both α and β) in *EGFR* MT NSCLCs [40], to our knowledge the activation of ER-α has never before been evaluated in *KRAS* MT ADs. Using two laboratory assays (RPPA and IHC) and two independent tissue study sets we herein demonstrate that the *KRAS* mutation is specifically associated with the activation of ER-α in a subgroup of patients with lung AD. A number of *in vitro* and *in vivo* studies have demonstrated that the use of the anti-estrogen therapy alone or in combination with an EGFR inhibitor has enhanced anti-proliferative activity. This indicates that the cross-talk between EGFR (and most likely its downstream substrates, which include RAS) and the ER modulates the progression and response to therapy of this subtype of lung cancer [34, 41-43]. While non-randomized phase II studies have shown response rates between 12-25% in non-stratified NSCLC patients treated with tamoxifene in combination with standard chemotherapy or anti-EGFR targeted agents [31, 44], so far these results have not been validated in randomized trials [31, 45]. Nonetheless, stratification based on the ER expression or activation has never been used to allocate patients to anti-ER targeted treatment. Stratification using genomic and proteomic markers may lead to the identification of patients that can benefit from the addition of an anti-ER agent to their therapeutic regimen.

A few limitations need to be addressed. Independently our two study sets showed that approximately 20% of *KRAS* MT ADs of the lung have significant activation of the ER-α, but the data were not significant when the conventional Allred score was applied. IHC scoring algorithms have not been optimized and standardized for ER-α in lung cancer, especially for the phosphorylated form. Although different scoring systems can be applied, consensus still needs to be reached [31]. Prospective analysis comparing the molecular profile with the clinical outcome in patients treated with anti-estrogen agents will be necessary to establish cut-points to identify patients that may benefit from anti-ER targeted treatment.

By exploring the signaling network of human lung ADs harbouring a *KRAS* mutation, this study identified new potential therapeutic targets for this group of patients which provide initial findings on the elucidation of a KRAS to in lung cancers. In particular the identification of ER-α, the AKT-mTOR network and multiple RTKs such as the HER family, c-Abl, Ret, and Ron, as potential *KRAS* MT oriented drug targets merits further evaluation using *in vitro* and *in vivo* studies.

## MATERIALS AND METHODS

### Tissue collection

Fifty-eight retrospective primary ADs of the lung collected from 2006 to 2012 at the H. Lee Moffitt Cancer Center and Research Institute (Tampa, FL) and at the S. Maria della Misericordia Hospital (Perugia, Italy) were included in this analysis. Only patients with surgically treated *EGFR* WT NSCLCs were included in the study. Enrolling institutions received Institutional Review Board approval for this study and informed consent was collected voluntarily from each patient before undergoing surgical removal of the primary tumor. For the signaling analysis, surgical specimens were snap-frozen within 30 minutes from collection and embedded in Optimal Cutting Temperature compound (OCT).

*EGFR* (Exons 18-21) and *KRAS* (Exons 1-2) mutation status was characterized at the enrolling institutions using Sanger sequencing protocols as previously described [46-48].

### Laser capture microdissection

Six to nine 8.0 μm sections were mounted on uncoated glass slides and stored at −80°C until processed further. For each specimen, one slide was stained with Hematoxylin (Sigma Aldrich, St. Louis, MO) and Eosin (Sigma Aldrich, St. Louis, MO) and examined by a certified pathologist (LL) to confirm the presence of malignant cells. LCM was used to isolate tumor epithelial cells from the surrounding microenvironment as previously described [49]. Briefly, slides were fixed in 70% ethanol, rinsed with deionized water, stained with Hematoxylin (Sigma Aldrich, St. Louis, MO) and Scott's Tap Water (Electron Microscopy Sciences, Hatfield, PA), and dehydrated in ethanol (70%, 95% and 100%) and xylene. Complete mini protease inhibitors (Roche Applied Science, Indianapolis, IN) were added to the 70% ethanol, deionized water, Hematoxylin, and Scott's Tap Water to preserve protein and phosphorylations from degradation.

Using the infrared laser of a Veritas microdissection system (Arcturus Bioscience, Mountain View, CA) 0.5-18 mm^2^ of malignant cells were collected from each sample on CapSure Macro LCM caps (Arcturus Bioscience, Mountain View, CA).

### Reverse phase protein microarray

Microdissected cells were stored at −80°C until lysed in a 1:1 solution of 2x Tris-Glycine SDS Sample buffer (Invitrogen Life Technologies, Carlsbad, CA) and Tissue Protein Extraction Reagent (Pierce, Rockford, IL) supplemented with 2.5% β-mercaptoethanol (Sigma, St. Louis, MO). Approximately 2.5 mm^2^ of microdissected tissue were lysed in 6 μl of buffer.

Cell lysates were immobilized onto nitrocellulose-coated slides (Grace Bio-labs, Bend, OR) using an Aushon 2470 arrayer (Aushon BioSystems, Billerica, MA). Each sample was printed in triplicate along with standard curves for internal quality control. Selected arrays were stained with Sypro Ruby Protein Blot Stain (Molecular Probes, Eugene, OR) following manufacturing instructions to quantify the amount of protein present in each sample [22].

Prior to antibody staining the remaining arrays were treated with Reblot Antibody Stripping solution (Chemicon, Temecula, CA) for 15 minutes at room temperature, washed with PBS and incubated for at least one hour in I-block (Tropix, Bedford, MA). Using an automated system (Dako Cytomation, Carpinteria, CA) arrays were first probed with 3% hydrogen peroxide, biotin blocking system (Dako Cytomation, Carpinteria, CA), and an additional serum free protein block (Dako Cytomation, Carpinteria, CA) to reduce unspecific binding between endogenous proteins and the detection system. Finally, arrays were probed with 150 antibodies, of which 114 targeted the phosphorylated sites of human kinases and downstream substrates (Supplementary Table 1). Antibodies were validated for their use on the array as previously described [50].

Biotinylated anti-rabbit (Vector Laboratories, Inc. Burlingame, CA) or anti-mouse secondary antibody (CSA; Dako Cytomation Carpinteria, CA) coupled with the Catalyzed Signal Amplification System (CSA; Dako Cytomation Carpinteria, CA), a commercially available tyramide-based avidin/biotin amplification kit, were employed to amplify the detection of the signal. Fluorescent detection was obtained using IRDye 680RD Streptavidin (LI-COR Biosciences, Lincoln, NE) according to the manufacturer's recommendation.

Antibody and Sypro Ruby stained slides were scanned on a Tecan laser scanner (TECAN, Mönnedorf, Switzerland) using the 620 nm and 580 nm weight length channel respectively. Images were analyzed with MicroVigene Software Version 5.1.0.0 (Vigenetech, Carlisle, MA) as previously described [22]. In brief, the software performs spot finding along with subtraction of the local background and unspecific binding generated by the secondary antibody. In addition, the program automatically normalizes each sample to the corresponding amount of protein derived from the Sypro Ruby stained slides and averages the triplicates. Intra and inter-assay reproducibility of the assay has been previously described [51]. *KRAS* status was assigned to each sample only after the molecular analysis was completed.

### Immunohistochemical analysis

A retrospective independent set of 90 NSCLCs collected at the S. Maria della Misericordia Hospital (Perugia, Italy) from 2002 to 2013 was used to further investigate, using immunohistochemistry (IHC), the activation level of the Estrogen Receptor α (ER-α). IHC was performed on 4 μm formalin-ﬁxed parafﬁn-embedded (FFPE) sections using a monoclonal antibody targeting the ER-α phosphorylation site S118 (Clone 16J4, Cell Signaling, dilution 1:50). The signal was detected using a biotin–free polymeric-horseradish peroxidase (HRP)-linker antibody conjugate system (Bond Polymer Refine Detection, Leica BioSystems, Newcastle, UK) with heat-induced epitope retrieval. Slides were stained using the Bond III automated immunostainer (Leica BioSystems Pty Ltd., Melbourne, VIC, Australia).

Semi-quantitative evaluation of the staining was performed independently by two pathologists (GB and AS). Only nuclear staining of the tumor cells was scored. Slides with controversial scoring were revisited by the pathologists and discussed until consensus was reached. *KRAS* status was revealed to the investigators only upon completion of analysis.

For each sample two variables were quantified: staining intensity and percentage of positive cells. Staining intensity was defined using an ordinal scale (0=no staining, 1= weak staining, 2= moderate staining, 3= strong staining) while percentage of positive cells was measured as a continuous variable (0-100%). Intensity values and percentage of stained cells were initially evaluated as independent variables. Subsequently the Allred Scoring System was applied to the data set as previously described [31]. In brief, the proportion of positive cells was first classified using an ordinal scale (0, no staining, 1= ≤ 1%, 2 = 2-10%, 3 = 11-33%, 4= 34-66%, 5= > 67%). The percentage of positive cells was then added to the value of the staining intensity to obtain a score ranging from 0 to 8. Finally all scores were further classified as negative (Allred score ≤ 1), weak (Allred score between 2 and 4), or strong (Allred score ≥ 5) [31].

### Statistical analysis

Non-parametric pairwise correlation analysis was used to explore the relationship between the phospho-isoforms of well-known KRAS downstream substrates (Raf S259, b-Raf S445, c-Raf S338, Mek 1/2 S217/221, and ERK 1/2 T202/Y204) and the expression/activation levels of the remaining 145 analytes measured by RPPA. Spearman's Rho correlation coefficients were calculated for *KRAS* MT and WT samples using JMP version 5.1 (SAS Institute Inc., SAS, Cary, NC). To control for potential errors associated with the multiple comparisons, and to identify only correlations with a strong statistical significance a Bonferroni correction was applied [52]. After the correction only *p* < 0.0003 were considered significant.

The expression/activation levels of the 150 analytes measured by RPPA between *KRAS* MT and WT cases were then compared. Based on whether the population distribution was normal, parametric two sample t-test or non-parametric Wilcoxon rank sum test were performed using R version 2.14.1 (R Development Core Team, Vienna, Austria). For the subgroup of analytes that reached statistical difference scatter plots with mean and standard error of the mean were generated with GraphPad Prism version 6 (GraphPad Software Inc., San Diego, CA).

Finally, for nominal and ordinal data (sex, stage, IHC intensity values, and Allred score) the proportion of cases across the different groups was assessed using the Pearson's Chi-square test. All significance levels, except for the correlation analysis, were set as *p*≤ 0.05.

## SUPPLEMENTARY MATERIAL TABLES


