# Supplementary material for: Functional signaling pathway analysis of lung adenocarcinomas identifies novel therapeutic targets for KRAS mutant tumors
Source: Oncotarget. 2015 Sep 30;6(32):32368–79. doi: 10.18632/oncotarget.5941 (PMC4741699; doi:10.18632/oncotarget.5941)
Supplement: Supplementary file 1 [file oncotarget-06-32368-s001.pdf]

Functional signaling pathway analysis of lung adenocarcinomas identifies novel therapeutic targets for *KRAS* mutant tumors

Supplementary Material

Supplementary table 1: List of 150 antibodies measured by RPPA.

Catalog Number	Antibody	Company	Dilution
9451	4E-BP1 (S65)	Cell Signaling	1:50
3661	Acetyl-CoA Carboxylase (S79)	Cell Signaling	1:50
9271	Akt (S473)	Cell Signaling	1:100
9275	Akt (T308)	Cell Signaling	1:100
3633	ALK (D5F3)	Cell Signaling	1:50
3341	ALK (Y1604)	Cell Signaling	1:50
4188	AMPKalpha1 (T172)	Cell Signaling	1:2000
4184	AMPKalpha1 (S485)	Cell Signaling	1:50
4181	AMPKBbeta1 (S108)	Cell Signaling	1:50
4431	a-Raf (S299)	Cell Signaling	1:100
9225	ATF-2 (T69/71)	Cell Signaling	1:1000
4331	ATP-Citrate Lyase (S454)	Cell Signaling	1:100
2772	Bax	Cell Signaling	1:200
2827	Bcl-2 (S70)	Cell Signaling	1:50
2762	Bcl-xL	Cell Signaling	1:500
2933	BIM	Cell Signaling	1:500
9434	b-Raf	Cell Signaling	1:100
2696	b-Raf (S445)	Cell Signaling	1:50
3532	Btk	Cell Signaling	1:50
2861	c-Abl (Y245)	Cell Signaling	1:100
9761	Caspase-6 cleaved (D162)	Cell Signaling	1:50
9501	Caspase-9 cleaved (D330)	Cell Signaling	1:50
9561	Catenin (beta) (S33/37/T41)	Cell Signaling	1:100
3570	CD44	Cell Signaling	1:50
2341	Chk1 (S345)	Cell Signaling	1:50
2665	Chk2 (S33/35)	Cell Signaling	1:50
9402	c-Myc	Cell Signaling	1:100
3313	Cofilin (S3)	Cell Signaling	1:100
2831	c-PLA2 (S505)	Cell Signaling	1:1000
9427	c-Raf (S338)	Cell Signaling	1:200

9191	CREB (S133)	Cell Signaling	1:100
2926	Cyclin D1	Cell Signaling	1:100
2921	Cyclin D1 (T286)	Cell Signaling	1:100
2232	EGFR	Cell Signaling	1:100
2238	EGFR (S1046/1047)	Cell Signaling	1:500
2234	EGFR (Y1068)	Cell Signaling	1:50
44-792	EGFR (Y1148)	Invitrogen	1:100
44-794	EGFR (Y1173)	Invitrogen	1:100
2235	EGFR (Y992)	Cell Signaling	1:50
3597	eIF2alpha (S51)	Cell Signaling	1:500
2441	eIF4G (S1108)	Cell Signaling	1:1000
9181	Elk-1(S383)	Cell Signaling	1:100
07-357	eNOS/eNOSIII (S116)	Upstate	1:500
2242	ErbB2/HER2	Cell Signaling	1:100
IMG-90189	ErbB2/HER2 (Y1248)	Imgenex	1:500
4561	ErbB3/HER3 (Y1197)	Cell Signaling	1:100
4791	ErbB3/HER3 (Y1289)	Cell Signaling	1:200
9102	ERK 1/2	Cell Signaling	1:200
9101	ERK 1/2 (T202/Y204)	Cell Signaling	1:1000
2511	Estrogen Receptor α (S118)	Cell Signaling	1:1000
3281	FAK (Y576/577)	Cell Signaling	1:200
9461	FKHR (S256)	Cell Signaling	1:100
9464	FKHR T24/FKHRL1 (T32)	Cell Signaling	1:200
06-953	FKHRL1 (S253)	Upstate	1:1000
Ab69758	Flt-3 Ligand	Abcam	1:500
3972	GRB2	Cell Signaling	1:1000
9331	GSK-3alpha/beta (S21/9)	Cell Signaling	1:1000
610958	HIF-1 alpha	BD	1:50
06-570	Histone H3 (S10) Mitosis Marker	Upstate	1:200
07-145	Histone H3 (S28)	Upstate	1:1000
2406	HSP27 (S82)	Cell Signaling	1:100
SPA-810	HSP70	Stressgen	1:100
3488	HSP90a (T5/7)	Cell Signaling	1:100
3021	IGF-1 Rec (Y1131)/Insulin Rec (Y1146)	Cell Signaling	1:500
9246	IkappaB-alpha (S32/36)	Cell Signaling	1:100
MAB306	IL-7 Receptor	R&D	1:100
2386	IRS-1 (S612)	Cell Signaling	1:200
3331	Jak1 (Y1022-1023)	Cell Signaling	1:50
4406	Jak2 (Y1007)	Cell Signaling	1:200

44-850	Lck (Y505)	Biosource	1:50
3051	LKB1 (S428)	Cell Signaling	1:100
2741	MARCKS (S152/156)	Cell Signaling	1:200
3521	MDM2 (S166)	Cell Signaling	1:100
9128	MEK1 (S298)	Cell Signaling	1:2000
9121	MEK1/2 (S217/221)	Cell Signaling	1:500
3126	Met (Y1234/1235)	Cell Signaling	1:200
9594	MSK1 (S360)	Cell Signaling	1:50
2972	mTOR	Cell Signaling	1:200
2971	mTOR (S2448)	Cell Signaling	1:100
3031	NF-kappa B p65 (S536)	Cell Signaling	1:100
71-7700	p27 (T187)	Zymed	1:200
610242	p27/kip1	BD	1:100
9211	p38 MAP kinase (T180/Y182)	Cell Signaling	1:100
9282	p53	Cell Signaling	1:5000
9284	p53 (S15)	Cell Signaling	1:1000
9202	p70S6 Kinase	Cell Signaling	1:100
07-018	p70S6 Kinase (T412)	Upstate	1:500
9208	p70S6 Kinase (S371)	Cell Signaling	1:50
9205	p70S6 Kinase (T389)	Cell Signaling	1:100
9341	p90RSK (S380)	Cell Signaling	1:400
9344	p90RSK (T359/S363)	Cell Signaling	1:200
2601	PAK1 (T423)/PAK2 (T402)	Cell Signaling	1:100
2607	PAK2 (S20)	Cell Signaling	1:100
9541	PARP cleaved D214	Cell Signaling	1:100
07-021	PDGF Receptor beta (Y716)	Upstate	1:250
3161	PDGF Receptor beta (Y751)	Cell Signaling	1:50
3061	PDK1 (S241)	Cell Signaling	1:200
1683-1	PI3-Kinase p110a	Upstate	1:50
4252	PI3-Kinase p110gamma	Cell Signaling	1:100
4781	PKA C (T197)	Cell Signaling	1:200
06-822	PKC alpha (S657)	Cell Signaling	1:1000
9376	PKC delta/theta (S643/676)	Cell Signaling	1:500
9377	PKC theta (T538)	Cell Signaling	1:100
9378	PKC zeta/lambda (T410/403)	Cell Signaling	1:50
2821	PLCgamma1 (Y783)	Cell Signaling	1:100
3872	PLCgamma2	Cell Signaling	1:200
2039	PP2A A subunit	Cell Signaling	1:1000
4953	PP2A B subunit	Cell Signaling	1:1000

3171	Progesterone Receptor (S190)	Cell Signaling	1:50
9552	PTEN	Cell Signaling	1:50
9551	PTEN (S3800)	Cell Signaling	1:500
3291	Pyk2 (Y402)	Cell Signaling	1:200
9421	Raf (S259)	Cell Signaling	1:100
05-516	Ras	Upstate	1:200
3321	Ras-GRF1 (S916)	Cell Signaling	1:50
3590	Rb (S780)	Cell Signaling	1:2000
3221	Ret (Y905)	Cell Signaling	1:100
5176-1	Ron (Y1353)	Epitomics	1:1000
4856	S6 Ribosomal Protein (S235/236)	Cell Signaling	1:200
2215	S6 Ribosomal Protein (S240/244)	Cell Signaling	1:1000
9251	SAPK/JNK (T183/Y185)	Cell Signaling	1:100
9155	SEK1/MKK4 (S80)	Cell Signaling	1:50
5599	SGK1 (S78)	Cell Signaling	1:100
07-206	Shc (Y317)	Upstate	1:200
3941	SHIP1 (Y1020)	Cell Signaling	1:50
44-558	SHP2 (Y580)	Biosource	1:500
9511	Smad1/Smad5/Smad8 (S/S/S)	Cell Signaling	1:50
3104	Smad2 (S245/250/255)	Cell Signaling	1:100
3101	SMAD2 (S465/467)	Cell Signaling	1:200
3950	SOCS1	Cell Signaling	1:50
2923	SOCS3	Cell Signaling	1:50
2101	Src family (Y416)	Cell Signaling	1:200
2105	Src (Y527)	Cell Signaling	1:200
9171	Stat1 (Y701)	Cell Signaling	1:1000
441	Stat2 (Y690)	Cell Signaling	1:100
9134	Stat3 (S727)	Cell Signaling	1:100
9145	Stat3 (Y705)	Cell Signaling	1:100
5267	Stat4 (Y693)	Cell Signaling	1:100
9351	Stat5 (Y694)	Cell Signaling	1:50
9361	Stat6 (Y641)	Cell Signaling	1:100
2808	Survivin (71G4)	Cell Signaling	1:100
3736	TNF-R1	Cell Signaling	1:50
9321	Tyk2 (Y1054-1055)	Cell Signaling	1:500
4657	VAV-1	Cell Signaling	1:7500
2471	VEGFR 2 (Y951)	Cell Signaling	1:200
2474	VEGFR2 (Y996)	Cell Signaling	1:100
2478	VEGFR 2 (Y1175)	Cell Signaling	1:50

2738	VHL	Cell Signaling	1:1000
2530	Wnt5a-B	Cell Signaling	1:100
2701	Zap-70 Y319-Syk (Y352)	Cell Signaling+	1:400

Supplementary table 2: Spearman's Rho correlation coefficients for endpoints statistically significant after Bonferroni correction in WT tumors.

Raf S259 correlations		
**Analyte**	**Spearman Rho**	**pValue**
**RTKs**		
EGFR (S1046/1047)	0.7786	<.0001
**MAPK pathway**		
b-Raf (S445)	0.8292	<.0001
ERK 1/2 (T202/Y204)	0.7519	0.0001
Ras-GRF1 (S916)	0.9023	<.0001
**Erk 1/2 downstream effectors**		
CREB (S133)	0.8146	<.0001
p90RSK (S380)	0.7278	0.0003
PAK2 (S20)	0.8824	<.0001
PKC delta/theta (S643/676)	0.7366	0.0002
**Akt/mTOR pathway**		
AMPKalpha1 (S485)	0.7474	0.0002
FKHR (S256)	0.7454	0.0002
GSK-3alpha/beta (S21/9)	0.7684	<.0001
mTOR (S2448)	0.8391	<.0001
**Other**		
ATP-Citrate Lyase (S454)	0.8135	<.0001

B-Raf S445 correlations		
**Analyte**	**Spearman Rho**	**pValue**
**MAPK pathway**		
Raf (S259)	0.8292	<.0001
**Erk 1/2 downstream effectors**		
PAK2 (S20)	0.8473	<.0001
PKC delta/theta (S643/676)	0.745	<.0001
**Akt/mTOR pathway**		
GSK-3alpha/beta (S21/9)	0.7051	0.0001
**Other**		
Acetyl-CoA (S79)	0.7081	0.0001
ATP-Citrate Lyase (S454)	0.7099	0.0001

c-Raf S338 correlations		
**Analyte**	**Spearman Rho**	**pValue**

**MAPK pathway**		
ERK 1/2 (T202/Y204)	0.6797	0.0003
**Akt/mTOR pathway**		
GSK-3alpha/beta (S21/9)	0.678	0.0003

**Mek 1-2 S217/221 correlations**		
**Analyte**	**Spearman Rho**	**pValue**
**RTKs**		
Met (Y1234/1235)	0.7287	<.0001
**MAPK pathway**		
SAPK/JNK(T183/Y185)	0.7171	<.0001
Stat5 (Y694)	0.7774	<.0001
**Erk 1/2 downstream effectors**		
cPLA2 (S505)	0.8256	<.0001
Smad2 (S245/250/255)	0.6759	0.0003
**Other**		
cPLA2 (S505)	0.7748	<.0001

**pERK 1-2 correlations**		
**Analyte**	**Spearman Rho**	**pValue**
**RTKs adaptor proteins**		
Shc (Y317)	0.7261	<.0001
Src (Y527)	0.6835	0.0002
**MAPK pathway**		
c-Raf (S338)	0.6797	0.0003
p38 MAPK (T180/Y182)	0.7287	<.0001
Raf (S259)	0.7519	0.0001
Ras-GRF1 (S916)	0.7070	0.0001
SAPK/JNK (T183/Y185)	0.6945	0.0002
Stat5 (Y694)	0.7070	0.0001
**Erk 1/2 downstream effectors**		
p90RSK (S380)	0.7374	<.0001
**Akt/mTOR pathway**		
AMPKalpha1 (S485)	0.7226	<.0001
LKB1 (S428)	0.7096	0.0001
mTOR (S2448)	0.8078	<.0001
p70 S6 Kinase (T412)	0.7502	<.0001
**Other**		
MDM2 (S166)	0.6730	0.0003

Supplementary table 3: Spearman's Rho correlation coefficients for endpoints statistically significant after Bonferroni correction in MT tumors.

Raf S259 correlations		
**Analyte**	**Spearman Rho**	**pValue**
**RTKs**		
c-Abl (Y245)	0.6290	<.0001
Ron (Y1353)	0.6461	<.0001
**MAPK pathway**		
b-Raf (S445)	0.7415	<.0001
c-Raf (S338)	0.6934	<.0001
Erk total	0.6927	<.0001
MEK 1/2 (S217/221)	0.6260	<.0001
Ras-GRF1 (S916)	0.7945	<.0001
**Erk 1/2 downstream effectors**		
p90RSK (S380)	0.6908	<.0001
PAK1 (T423)/PAK2 (T402)	0.6296	<.0001
PAK2 (S20)	0.7657	<.0001
PKC delta/theta (S643/676)	0.7674	<.0001
PKC theta (T538)	0.7396	<.0001
Stat5 (Y694)	0.6514	<.0001
**Akt/mTOR pathway**		
4E-BP1	0.6633	<.0001
AMPK alpha1 (T172)	0.6621	<.0001
AMPKalpha1 (S485)	0.5892	0.0003
eNOS/eNOSIII (S116)	0.6678	<.0001
GSK-3alpha/beta (S21/9)	0.7219	<.0001
LKB1 (S428)	0.6780	<.0001
mTOR total	0.6264	<.0001
mTOR (S2448)	0.6424	<.0001
NF-kappa B p65 (S536)	0.7469	<.0001
p70 S6 Kinase (T412)	0.6336	<.0001
PTEN	0.5914	0.0003
PTEN (S380)	0.6704	<.0001
**Other**		
Acetyl-CoA (S79)	0.6497	<.0001
ATP-Citrate Lyase (S454)	0.6979	<.0001
Histone H3 (S28)	0.7961	<.0001

B-Raf S445 correlations		
**Analyte**	**Spearman Rho**	**pValue**
**RTKs**		
PDGF Receptor beta (Y751)	0.6619	<.0001
Ret (Y905)	0.5834	0.0003
**MAPK pathway**		
c-Raf (S338)	0.7608	<.0001
Erk total	0.5882	0.0003
MEK 1/2 (S217/221)	0.6755	<.0001
HSP27 (S82)	0.6625	<.0001
Raf (S259)	0.7415	<.0001
Ras-GRF1 (S916)	0.7576	<.0001
**Erk 1/2 downstream effectors**		
CREB (S133	0.6437	<.0001
PAK1 (T423)/PAK2 (T402)	0.6796	<.0001
PAK2 (S20)	0.7375	<.0001
PKC delta/theta (S643/676)	0.7619	<.0001
Smad1/Smad5/Smad8 S/S	0.5779	0.0003
**Akt/mTOR pathway**		
4E-BP1 (S65)	0.6741	<.0001
eNOS/eNOSIII (S116)	0.7623	<.0001
GSK-3alpha/beta (S21/9)	0.6322	<.0001
LKB1 (S428)	0.6345	<.0001
mTOR (S2448)	0.6096	0.0001
NF-kappa B p65 (S536)	0.5920	0.0002
PTEN	0.6628	<.0001
PTEN (S380)	0.7074	<.0001
**Other**		
Acetyl-CoA (S79)	0.7304	<.0001
ATP-Citrate Lyase (S454)	0.7959	<.0001
Histone H3 (S28)	0.7906	<.0001
p53 (S15)	0.6535	<.0001

C-Raf S338 correlations		
**Analyte**	**Spearman Rho**	**pValue**
**RTKs**		
ErbB3/Her3 (Y1197)	0.6098	0.0001
PDGF Receptor beta (Y751)	0.6130	0.0001
**MAPK pathway**		
B-Raf (S445)	0.7608	<.0001
MEK 1/2 (S217/221)	0.5939	0.0002

Raf (S259)	0.6934	<.0001
Ras-GRF1 (S916)	0.7337	<.0001
Stat5 (Y694)	0.5867	0.0003
**Erk 1/2 downstream effectors**		
CREB (S133)	0.7463	<.0001
Elk-1 (S383)	0.7125	<.0001
p90RSK (S380)	0.7832	<.0001
PAK2 (S20)	0.6675	<.0001
ATF-2 (T69/71)	0.7698	<.0001
**Akt/mTOR pathway**		
eNOS/eNOSIII (S116)	0.6480	<.0001
FKHR T24/FKHRL1 (T32)	0.5872	0.0003
GSK-3alpha/beta (S21/9)	0.7029	<.0001
LKB1 (S428)	0.7834	<.0001
mTOR total	0.5990	0.0002
mTOR (S2448)	0.7288	<.0001
NF-kappa B p65 (S536)	0.5869	0.0003
p70 S6 Kinase (T412)	0.6994	<.0001
p70 S6 Kinase (T389)	0.6033	0.0002
PTEN (S380)	0.6218	<.0001
**Other**		
MDM2 (S166)	0.5891	0.0002
Acetyl-CoA (S79)	0.7031	<.0001
ATP-Citrate Lyase (S454)	0.7817	<.0001
Histone H3 (S28)	0.7090	<.0001
CD44	0.5861	0.0003

Mek 1-2 S217/221 correlations		
**Analyte**	**Spearman Rho**	**pValue**
**RTKs**		
c-Abl (Y245)	0.6712	<.0001
ErbB2/Her2 (Y1248)	0.6883	<.0001
EGFR (S1046/1047)	0.5861	0.0003
EGFR (Y1173)	0.6605	<.0001
Met (Y1234/1235)	0.5753	0.0003
PDGF Receptor beta (Y751)	0.6009	0.0002
**RTK adaptor proteins**		
Shc (Y317)	0.5964	0.0002
**MAPK pathway**		
B-Raf (S445)	0.6755	<.0001
c-Raf (S338)	0.5939	0.0002

ERK 1/2 (T202/Y204)	0.5828	0.0003
Raf (S259)	0.6260	<.0001
Ras-GRF1 (S916)	0.8111	<.0001
SAPK/JNK (T183/Y185)	0.6361	<.0001
Stat5 (Y694)	0.7653	<.0001
Elk-1 (S383)	0.6706	<.0001
p90RSK (S380)	0.5719	0.0003
PAK1 (T423/PAK2 T402)	0.7891	<.0001
PAK2 (S20)	0.6755	<.0001
PKC theta (T538)	0.6695	<.0001
Smad1/Smad5/Smad8 S/S	0.5709	0.0003

**pERK 1-2 correlations: Mutant**		
**Analyte**	**Spearman Rho**	**pValue**
**RTKs**		
EGFR (Y1068)	0.6430	<.0001
EGFR (Y1173)	0.6593	<.0001
**RTK adaptor proteins**		
Shc (Y317)	0.7038	<.0001
**MAPK pathway**		
MEK 1/2 (S217/221)	0.5828	0.0003
SAPK/JNK T183/Y185)	0.8196	<.0001
Stat5 (Y694)	0.6543	<.0001
Tyk2 (Y1054_1055)	0.6593	<.0001
**Erk substrates**		
Elk-1 (S383)	0.7819	<.0001
FAK (Y576/577)	0.5816	0.0003
p90RSK (S380)	0.6062	0.0001
Smad2 S245/250/255	0.5910	0.0002
**Akt/mTOR pathway**		
Akt (S473)	0.7018	<.0001
GSK-3alpha/beta (S21/9)	0.6471	<.0001
mTOR (S2448)	0.6491	<.0001
**Other**		
ATP-Citrate Lyase (S454)	0.5871	0.0003
cPLA2 (S505)	0.6317	<.0001
MDM2 (S166)	0.7400	<.0001
